# Response of patients with acute respiratory failure caused by COVID-19 to awake-prone position outside the intensive care unit based on pulmonary involvement

**DOI:** 10.6061/clinics/2021/e3368

**Published:** 2021-11-25

**Authors:** João Manoel Silva, Ricardo Esper Treml, Pamela Cristina Golinelli, Miguel Rogério de Melo Gurgel Segundo, Pedro Ferro L. Menezes, Julilane Daniele de Almeida Umada, Ana Paula Santana Alves, Renata Peres Nabeshima, André dos Santos Carvalho, Talison Silas Pereira, Elaine Serafim Sponton

**Affiliations:** I Departamento de Anestesia, Faculdade de Medicina FMUSP, Universidade de Sao Paulo, Sao Paulo, SP, BR.; II Programa de Pos-Doutorado em Anestesiologia, Faculdade de Medicina FMUSP, Universidade de Sao Paulo, Sao Paulo, SP, BR.; III Department of Anesthesia, Critical Care and Pain Medicine, University of Jena, Jena, Germany.; IV Departamento de Enfermagem, Hospital do Servidor Publico Estadual (IAMPSE), Sao Paulo, SP, BR.; V 11 Health Care Services, Sao Paulo, SP, BR.

**Keywords:** COVID-19, Prone Position, Noninvasive Ventilation, Critical Care Outcome, Respiratory Insufficiency

## Abstract

**OBJECTIVES:**

Since there are difficulties in establishing effective treatments for COVID-19, a vital way to reduce mortality is an early intervention to prevent disease progression. This study aimed to evaluate the performance of patients with COVID-19 with acute hypoxic respiratory failure according to pulmonary impairment in the awake-prone position, outside of the intensive care unit (ICU).

**METHODS:**

A prospective observational cohort study was conducted on COVID-19 patients under noninvasive respiratory support. Clinical and laboratory data were obtained for each patient before the treatment and after they were placed in the awake-prone position. To identify responders and non-responders after the first prone maneuver, receiver operating characteristic curves with sensitivity and specificity of the PaO_2_/FiO_2_ and SpO_2_/FiO_2_ indices were analyzed. The maneuver was considered positive if the patient did not require endotracheal intubation for ventilatory assistance.

**RESULTS:**

Forty-eight patients were included, and 64.6% were categorized as responders. The SpO_2_/FiO_2_ index was effective for predicting endotracheal intubation in COVID-19 patients regardless of lung parenchymal damage (area under the curve 0.84, cutoff point 165, sensitivity 85%, specificity 75%). Responders had better outcomes with lower hospital mortality (hazard ratio [HR]=0.107, 95% confidence interval [CI]: 0.012-0.93) and a shorter length of stay (median difference 6 days, HR=0.30, 95% CI: 0.13-0.66) after adjusting for age, body mass index, sex, and comorbidities.

**CONCLUSIONS:**

The awake-prone position for COVID-19 patients outside the ICU can improve oxygenation and clinical outcomes regardless of the extent of pulmonary impairment. Furthermore, the SpO_2_/FiO_2_ index discriminates responders from non-responders to the prone maneuver predicting endotracheal intubation with a cutoff under or below 165.

## INTRODUCTION

Coronavirus disease (COVID-19) is caused by severe acute respiratory syndrome coronavirus 2 (SARS-CoV-2), and approximately 13.8% of COVID-19 patients become critical (1,2). Among patients with severe or critical diseases, acute respiratory distress syndrome (ARDS) is the most common presentation and main cause of admission to intensive care units (ICUs) (3). Since an effective drug treatment for COVID-19 is yet to be established, early recognition to prevent disease progression is vital to reduce mortality (4).

Prone positioning for patients with ARDS is beneficial for those on invasive mechanical ventilation (5). In addition, the prone position for spontaneously breathing patients has also been shown to improve lung heterogeneity and oxygenation (6).

In an attempt to mitigate the burden on ICUs with COVID-19 patients, the prone position for awake, spontaneously breathing patients may be a useful maneuver to improve oxygenation and avoid ICU transfers. Reports of prone position application for non-intubated, spontaneously breathing adult patients are limited (7,8). During the ongoing pandemic, this strategy has been used as rescue therapy, given the critical situation in intensive care services due to the high number of patients requiring invasive mechanical ventilation (9).

The role of the prone position and the potential effectiveness of this maneuver in COVID-19 patients who may progress to acute respiratory failure without requiring invasive mechanical ventilation are not fully understood, especially in patients outside the ICU (10). The first studies using awake-prone positioning showed a tendency toward clinical benefit after the implementation of this maneuver, resulting in improvement in oxygenation, reduction in treatment failure, and less need for intubation (6,11). However, an adequate clinical parameter for monitoring the patient’s response to the prone position has not been established. We hypothesize that the awake-prone position for spontaneously breathing patients with COVID-19 who need supplemental oxygen support improves oxygenation regardless of pulmonary involvement, could benefit them clinically, and possibly improve their outcome.

This study aimed to evaluate whether the prone position for COVID-19 patients without invasive mechanical ventilation can be used to prevent intubation for respiratory assistance, according to improvement in the PaO_2_/FiO_2_ and SpO_2_/FiO_2_ indices, despite pulmonary injury confirmed on chest computed tomography (CCT).

## METHODS

### Study design

A prospective observational cohort study was conducted in hospitalized COVID-19 patients with acute hypoxemic respiratory failure in a ward or emergency room without an indication for invasive mechanical ventilation, who required the maneuver as rescue therapy to improve blood oxygenation. This study was approved by the ethics committee of the hospital involved in the study in compliance with the Declaration of Helsinki and the STROBE guidelines for reporting observational studies (12) (Ethics Committee of the Hospital do Servidor Público Estadual de São Paulo-IAMPSE, 4.175.045/2020). All enrolled participants or their legal representatives provided written informed consent.

The study was conducted in an advanced public hospital during March and April 2021—the peak of the COVID-19 pandemic—in the state of São Paulo, Brazil. All patients were followed up until hospital discharge. In addition, laboratory data and clinical data such as the need for mechanical ventilation, mortality, and length of hospital stay were assessed.

The primary outcome was to evaluate the clinical response of awake-prone positioning according to improvement in the PaO_2_/FiO_2_ and SpO_2_/FiO_2_ indices. The secondary outcomes were hospital mortality and length of stay.

### Study population

This study included patients aged >18 years admitted outside the ICU (ward or emergency room) with a confirmed diagnosis of COVID-19 using the criteria established by the World Health Organization (WHO) (13) and receiving supplemental oxygen support or any type of noninvasive ventilatory support such as high-flow nasal cannula (HFNC) or continuous positive airway pressure (CPAP). The exclusion criteria for the awake-prone position maneuver were as follows: pregnancy, spinal instability, facial or pelvic fractures, thoracic surgery or unstable chest wall, delirium, inability to change position independently, and nausea and emesis.

### Data collection and outcome

All enrolled patients were observed until hospital discharge or death. Demographic data (age, sex, weight, body mass index, and comorbidities) were collected on the day of inclusion into the study. The following physiological variables were assessed during the hospital stay: respiratory rate, peripheral oxygen saturation (SpO_2_), heart rate, blood pressure, arterial blood gas analysis (ABG), and inspired oxygen fraction (FiO_2_) according to the respiratory support received. Pulmonary involvement was verified in all the patients on CCT at hospital admission. The degree of pulmonary involvement was quantitatively assessed by an independent radiologist (14). The categorization of lung parenchymal involvement was divided into two profiles: involvement <50% on CCT and involvement >50% on CCT (15).

Immediately before each pronation, patients’ SpO_2_, ABG, oxygen devices (CPAP or HFNC), oxygen flow (L/min), and clinical and laboratory data were evaluated. Documentation of the response per hour in pronation (SpO_2_, oxygen device, oxygen flow [L/min], blood pressure) was used to identify patients with a higher probability of benefit.

As a verified response, comfort or any alteration that could harm the patient, the maneuver was incorporated into clinical treatment for at least 1 hour and was adjusted during the day according to tolerance in the morning, afternoon, and at night. The patient was encouraged to recognize his/her discomfort at pressure points and to adjust as necessary, not remaining for more than 2 hours in the same position.

Thus, data on physiological variables collected during the first prone maneuver were used to categorize patients into responders and non-responders according to their progression and necessity of endotracheal intubation. Receiver operating characteristic (ROC) curves with sensitivity and specificity of the PaO_2_/FiO_2_ and SpO_2_/FiO_2_ oxygenation indices were analyzed to identify patients requiring endotracheal intubation; the maximum point was defined as the cutoff point to determine the need for tracheal intubation. Based on the best ROC curve, a cutoff point for responders and non-responders was defined. Independent of lung parenchyma involvement, the indices were tested for prediction of endotracheal intubation and compared in relation to clinical outcomes.

### Performance in the awake-prone position

Patients with respiratory symptoms requiring supplemental oxygen support or any noninvasive ventilatory support with a confirmed diagnosis of COVID-19 were enrolled, and an initial period of at least 1 hour three times a day in the prone position was indicated. The patient was laid face down supported by his or her arms and a pillow so that the oxygen supply would not be obstructed. Cushions were placed under the hips or legs as required for added comfort.

To verify initiation or maintenance of the awake-prone position were evaluated the mental status, mobility and onset of nausea and emesis. Supplemental oxygen support was adjusted as needed, and all patients were continuously monitored using a multiparametric monitor with oximetry evaluation. While monitoring the patient, the electrocardiogram electrodes were adjusted on the chest wall according to the patient’s position, and the SpO_2_ (continuous measurement) was maintained. Tolerance to maneuvering was defined as the total time that the patient remained in the awake-prone position and the degree of patient satisfaction (determined using a patient-satisfaction questionnaire). To minimize interruptions during the positioning of the face, comfort strategies were suggested to the patients such as using the bathroom as needed, keeping the nurses’ buzzer within reach, keeping the phone or other device in sight, and using a mobile phone or television as a distraction.

### Statistical analysis

Based on literature data (16) indicating that 50% of patients subjected to this strategy require tracheal intubation and considering a null hypothesis of 70% for intubation, with a type I error of 0.05 and a type II error of 0.2 (1- power), at least 44 patients would be necessary in this study; considering the possibility of follow-up losses, we estimated an inclusion of 50 patients.

The obtained data were inserted into an electronic database and analyzed using the statistical programs SPSS 27.0 and MedCalc 19.8.0.

Qualitative characteristics are described using absolute and relative frequencies, and the association within the groups was verified using the chi-square test or exact tests (Fisher's exact test or likelihood ratio test). Quantitative measurements are described using summary measures such as central tendency and dispersion measurements (mean and standard deviation, median and minimum and maximum values, or interquartile range) and compared between groups using Student’s t-test or the Mann-Whitney test according to the distribution pattern of the variables (Kirkwood and Sterne, 2006). The Kolmogorov-Smirnov test was used to evaluate the distribution patterns of continuous numerical variables. Variables obtained from repeated samples were compared using the Wilcoxon signed-rank test.

To determine the accuracy of the parameters with respect to patient responsiveness to the maneuver employed in the study and to determine the accuracy of these parameters in predicting endotracheal intubation, sensitivity and specificity tests of oxygenation indices were conducted after the first prone position of the patients. Thus, the ROC curve was created with values of sensitivity and specificity, and the maximum point was defined as a cutoff point to determine the absence of endotracheal intubation. Using this defined cutoff point, Kaplan-Meier curves of survival and hospital stay were generated through a stepwise Cox model adjusted for age, sex, comorbidities, and pulmonary involvement. All tests were two-tailed, and a *p*-value <0.05 was considered significant.

## RESULTS

**Figure 1:**
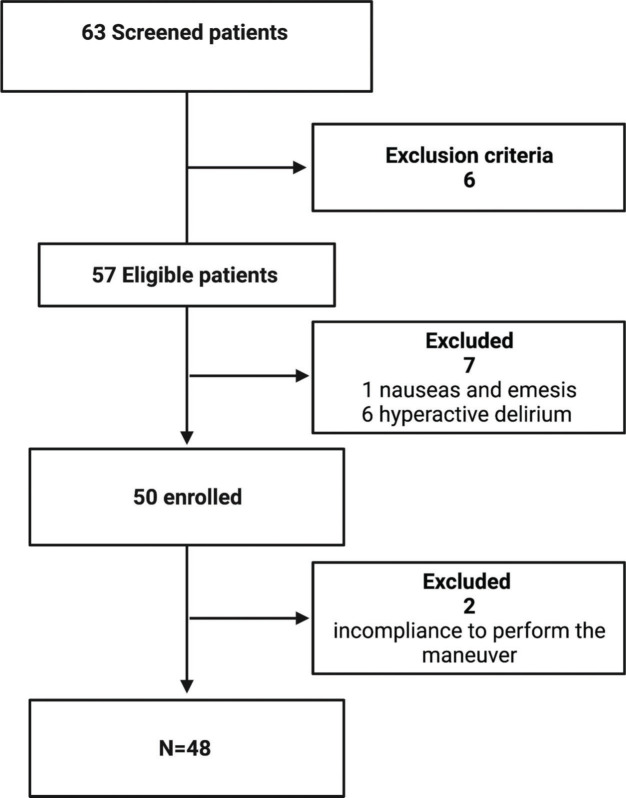
Study flow diagram.

Fifty patients were enrolled in this study. Owing to noncompliance with the institutional protocol during positioning, two patients were excluded (n=48 total) (Figure 1).

The median age was 61 years (interquartile range [IQR], 52 to 66.7), and the median body mass index was 28.7 kg/m^2^ (IQR, 24.5 to 32.9). Intubation and invasive ventilatory support were necessary in 33.3% of cases, with 63% having more than 50% pulmonary involvement and 37% having less than 50% on CCT. Overall, the mortality rate was 16.7% (Table 1).

The median duration of prone positioning was 2 hours, and the maximum duration was 4 hours. All patients who required invasive mechanical ventilation were intubated within 24 hours of the first prone positioning attempt.

When comparing all patients before and after awake-prone position with respect to the physiological and laboratory variables, regardless of lung involvement, there was a significant improvement in arterial oxygen saturation (SaO_2_) and SpO_2_ during prone position. In all patients with less than 50% pulmonary involvement, there was a significant improvement in arterial oxygen pressure (PaO_2_). In contrast, in all patients with pulmonary involvement higher than 50%, the prone maneuver showed a significant increase in mean blood pressure, improvement in arterial lactate levels, and increase in arterial bicarbonate and PaCO_2_ values (Table 2).

**Figure 2:**
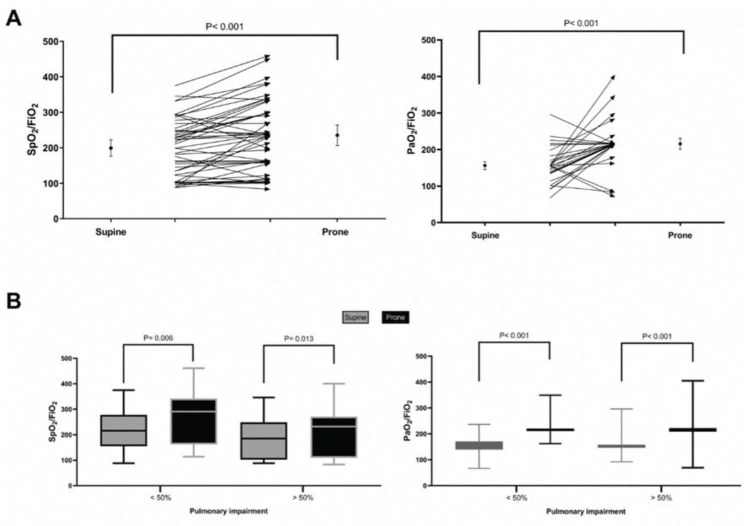
Comparison of SpO_2_/FiO_2_ and PaO_2_/FiO_2_ in the supine and prone positions (A); comparison of SpO_2_/FiO_2_ and PaO_2_/FiO_2_ in the supine and prone positions based on pulmonary involvement (B).

The medians of the SpO_2_/FiO_2_ and PaO_2_/FiO_2_ indices were significantly higher in the prone than in the supine position. The median paired difference between supine (205; IQR 123.5-252.5) and prone (235; IQR 159-301.5) SpO_2_/FiO_2_ was 20.5, *p*<0.001, and the median paired difference between supine (156.3; IQR 146. 6-156. 3) and prone (215.7; IQR 215. 7-252.5) PaO_2_/FiO_2_ was 59.4, *p*<0.001. Regardless of pulmonary impairment, were observed significant improvements on the indices SpO_2_/FiO_2_ and PaO_2_/FiO_2_ in the awake-prone position in comparison to supine position (Figure 2).

**Figure 3:**
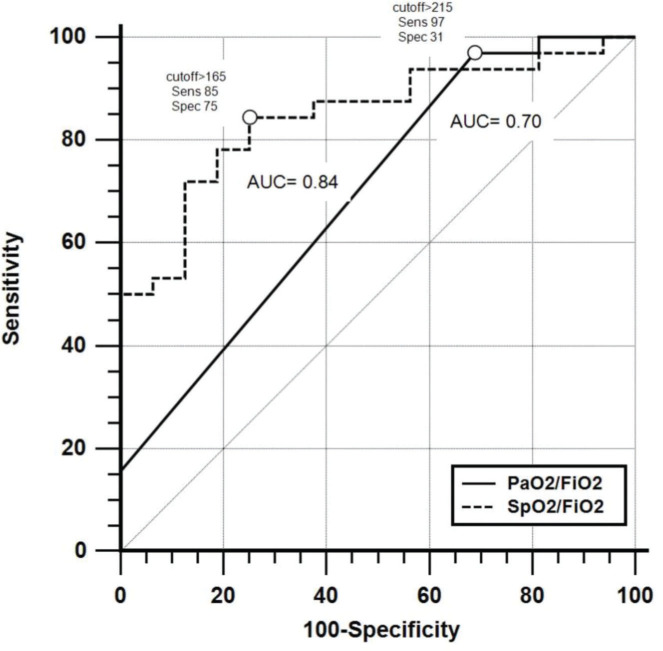
SpO_2_/FiO_2_ and PaO_2_/FiO_2_ ROC curves for predicting the need for endotracheal intubation.

The ability to discriminate between patients not requiring intubation was greater when considering the prone position’s SpO_2_/FiO_2_ index (AUC 0.84, cutoff point 165, sensitivity 85%, and specificity 75%) rather than considering the PaO_2_/FiO_2_ index (AUC 0.70, cutoff point 215, sensitivity 97%, and specificity 31%) (Figure 3).

**Figure 4:**
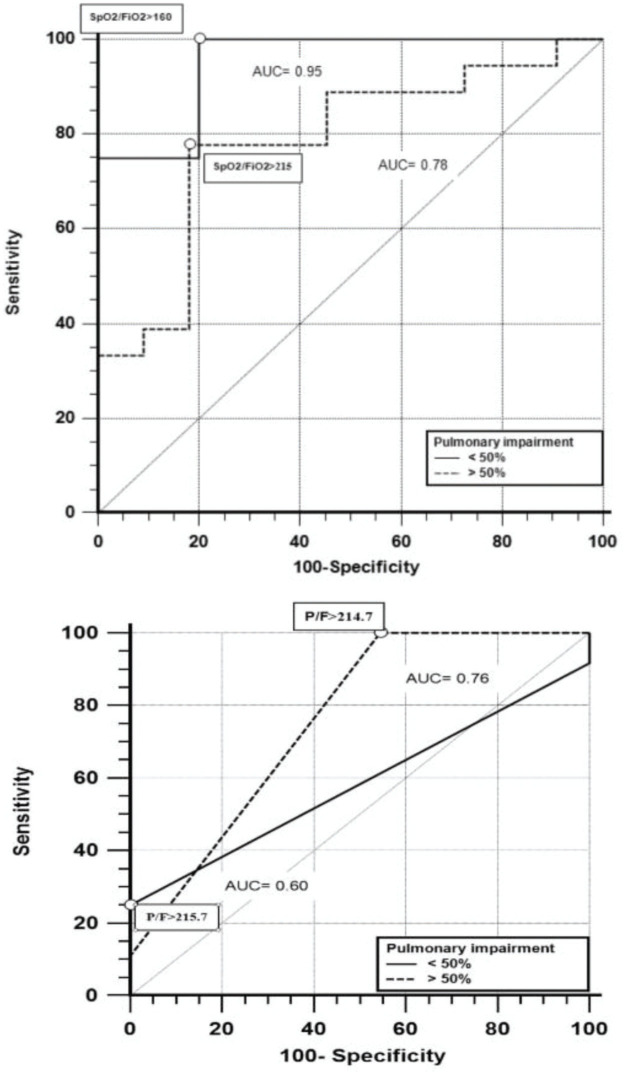
ROC curve analysis of the effectiveness of using SpO_2_/FiO_2_ and PaO_2_/FiO_2_ for predicting the need for endotracheal intubation based on pulmonary impairment. The top figure represents SpO_2_/FiO_2_, and the bottom figure represents PaO_2_/FiO_2_.

The best performance was achieved with the SpO_2_/FiO_2_ index mainly in patients with less than 50% pulmonary impairment, whereas that achieved with the PaO_2_/FiO_2_ index was mainly in patients with more than 50% pulmonary impairment (Figure 4).

**Figure 5:**
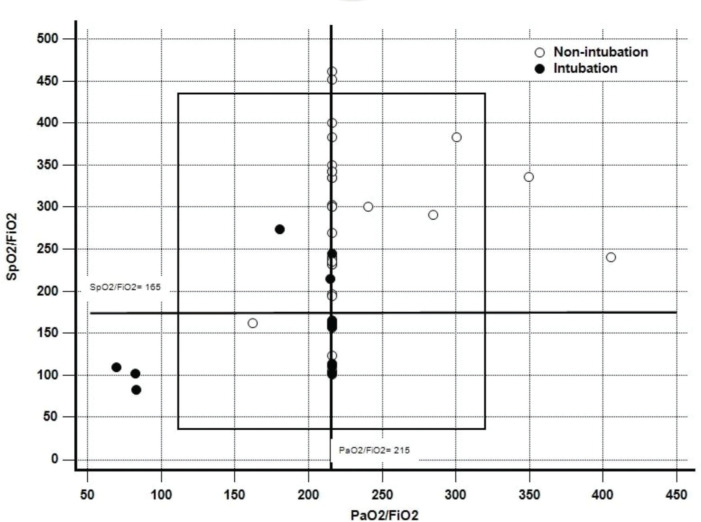
Relationship between patients who required endotracheal intubation and the SpO2/FiO2 and PaO2/FiO2 indices, and points of best accuracy of these indices.

Therefore, considering the best oxygenation index to discriminate endotracheal intubation, 64.6% of patients were responders (SpO2/FiO2 >165 prone) (Figure 5).

**Figure 6:**
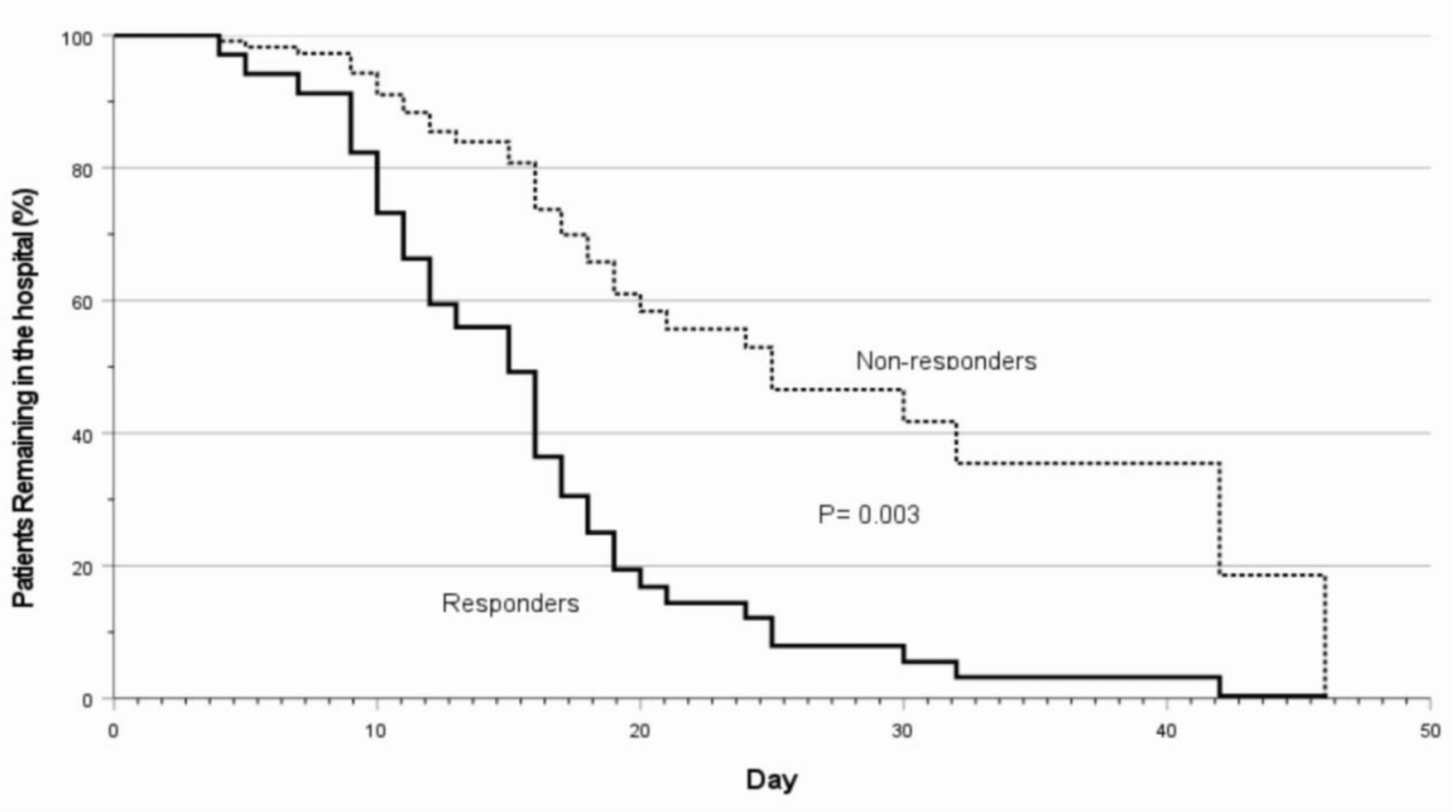
Kaplan-Meier analysis adjusted using a Cox regression model according to age, sex, pulmonary impairment, and comorbidities of responders and non-responders.

Hospital mortality in responders had a lower hazard ratio (HR) of 0.30 (CI 95%; 0.14-0.66), *p*=0.043 adjusted for age, sex, pulmonary impairment, and comorbidity, and they also had a shorter median hospital stay at 13 days (10-18.7) days *vs.* 19 (15.7-26) days, with a higher chance of hospital discharge HR=3.31 (95% CI, 1.50-7.31), *p*=0.003 (Figure 6).

In addition, responders had a shorter length of ICU stay (LOS-ICU) than non-responders, but the difference was not significant (*p*=0.113, 7.5 [6.0-8.5] days *vs.* 15.5 [7.5-19.0] days).

## DISCUSSION

In this prospective observational cohort study, we investigated the use of prone positioning in patients with COVID-19 who breathed spontaneously, were not intubated, and were admitted outside the ICU. The main finding of our study was that 66.4% of these patients were categorized as responders to the prone maneuver, improving their oxygenation parameters and avoiding endotracheal intubation. The SpO_2_/FiO_2_ index was used to categorize patients into responders and non-responders and to predict the need for endotracheal intubation for patients with an index below 165. Moreover, prone positioning in the responder group reduced the hazard of death by 70% (HR=0.30), when adjusted for age, sex, pulmonary impairment, and comorbidities. Responders also had a shorter median day hospital stay of 13 (10-18.7) days *vs.* 19 (15.7-26) days than non-responders, with a higher chance of hospital discharge (HR=3.31). Furthermore, the maneuver was effective in improving oxygenation and laboratory and physiological parameters.

Although some studies (17,18) have failed to determine better outcomes using awake-prone position, it was shown that noninvasively ventilated COVID-19 patients in different settings had lower mortality (19,20) and endotracheal intubation rates (11,19). Similarly, we found better chances of survival in patients considered responders to the prone position and found lower endotracheal intubation rates and hospital stay in this group of patients admitted outside the ICU. Another prospective study (16) on patients with ARDS caused by SARS-CoV-2 submitted to an awake-prone position outside the ICU setting demonstrated the applicability of the maneuver and the improvement in oxygenation through this position. The current study showed a significant improvement in the PaO_2_/FiO_2_ index in the prone position and additionally demonstrated improvement in oxygenation through the SpO2/FiO2 index in comparison with the supine position. SpO_2_/FiO_2_ values between 150 and 170 have been proposed in the literature as predictors of failure to respond to noninvasive ventilation and predictors of transfer to the ICU among patients with ARDS (21,22). An SpO_2_/FiO_2_ index >160 based on the absence of endotracheal intubation/mechanical ventilation defined patients as responders in this study. Thus, these results support the utility of the awake-prone position for patients outside the ICU setting as a possible maneuver that could reduce mortality and length of hospital stay.

It is important to note that there is evidence (20) demonstrating the benefits of early prone positioning for improving oxygenation and patient outcomes. To corroborate this, the present study was performed on outside-ICU patients who were still undergoing the first prone maneuver, that is, at hospital admission, and therefore, the clinical benefit may have resulted. In addition, a specific care protocol was used when patients were in the prone position that guaranteed a median time of 2 hours in the prone position, however, the optimal duration of prone positioning is unknown. Although the mean duration of prone positioning was 17 hours per day in the prone group compared with 0 hours in the supine group in the first study that reported a mortality benefit in mechanically ventilated patients (5), in another study (18), the median duration of awake-prone positioning per day was 9.0 hours in the prone group and 3.4 hours in the control group, and this difference was not large enough to decrease the rate of intubation.

Previous studies on spontaneously breathing patients without (8,23) and with COVID-19 (9,24,25) tested the strategy of awake-prone positioning and showed improvement in oxygenation, but no study tested the efficacy of the awake-prone position based on pulmonary involvement observed on CCT. In our study, we found a significant improvement in oxygenation regardless of the degree of pulmonary parenchyma involvement. This was reflected in the improvement in PaO_2_ and SpO_2_ when compared with those in the supine position. Interestingly, in patients with pulmonary involvement >50%, improvements in hemodynamic parameters such as arterial lactate levels and increased mean blood pressure were noted. This finding supports those in other studies that showed improvement in hemodynamic parameters in patients with ARDS in the prone position (26,27). However, there was a significant increase in PaCO_2_ and arterial HCO_3_ levels in this group. This may have been owing to the greater degree of involvement of the pulmonary parenchyma, leading to worsening of gas exchange, causing hypercapnia, respiratory acidosis, and compensatory elevation of HCO_3_ (28,29).

Another important finding of this study is a clearer definition of patients considered responders to the awake-prone position. Identification of the best response is much simpler by measuring oxygen saturation with a pulse oximeter. The use of the noninvasive SpO_2_/FiO_2_ index for COVID-19 patients proved to be a safe and sensitive tool for hypoxia screening in non-ICU settings (30). Moreover, together with the PaO_2_/FiO_2_ ratio, the SpO_2_/FIO_2_ index has also been used as a parameter to predict the failure of noninvasive support in patients with ARDS (22,31) and can discriminate extubation failure in COVID-19 patients (32). In our study, the SpO_2_/FiO_2_ index was effective in predicting endotracheal intubation for COVID-19 patients regardless of pulmonary parenchyma, with higher sensitivity and specificity (AUC 0.84, cutoff point 165, sensitivity 85%, and specificity 75%). The constancy of the SpO_2_/FiO_2_ index >165 after prone positioning indicated a reduced risk of endotracheal intubation, in turn, reducing the risk of mortality and ensuring earlier discharge. Several studies (33,34) have shown that COVID-19 patients who require endotracheal intubation have worse clinical prognoses, and even those who survive have unfavorable recovery. Therefore, determining a simpler and effective way to evaluate the best response in awake patients in the prone position could help make more accurate decisions to continue with the maneuver at this stage. Additionally, it was observed that greater pulmonary involvement did not interfere with the evaluation of the index, thus demonstrating the efficacy of this measure.

However, the data from this study should be interpreted with caution because of the study limitations. The study was conducted at a single center; hence, extrapolation of its results to other regions should be performed with caution. Other limitations to be considered are the small number of cohort groups and the lack of a control group. Further comparisons between different methods of supplemental oxygenation and noninvasive ventilation (HFNC and CPAP) are needed to precisely determine which population would best benefit from the prone position. In addition, endotracheal intubation criteria have not been uniformly defined and protocolized, limiting the definition of our results. Finally, individual responses could not be determined, limiting the possibility of analyzing predictive effects for intubation in specific subpopulations. Nevertheless, our current data showed that responders who had improved blood oxygenation through the awake-prone position may have a lower risk of endotracheal intubation, with possible improved outcomes shown through better chances of survival and hospital discharge.

## CONCLUSION

Self-proning outside the ICU was effective in improving oxygenation in COVID-19 patients regardless of pulmonary impairment. Patients with an SpO_2_/FiO_2_ index >165 were defined as responders who less likely required endotracheal intubation. Patients considered responders had better outcomes indicated by a reduced risk of death and a higher chance of hospital discharge.

### Highlights

- Patients with COVID-19 who needed supplemental oxygen supply or any noninvasive ventilation support outside the ICU were placed in the prone position.
- Patients categorized as responders to the prone position (SpO_2_/FiO_2_>150 and not requiring endotracheal intubation) had better outcomes and a lower risk of mortality and were discharged earlier from the hospital.
- SpO_2_/FiO_2_ levels can be used to determine the need for endotracheal intubation (cutoff 165) with higher sensitivity and specificity.

## AUTHOR CONTRIBUTIONS

All authors contributed equally to this study. Silva Junior JM supervised the study. The data were collected by Golinelli PC, Gurgel Segundo MR de M, Umada JD de A, Alves APS, Nabeshima RP, Menezes PFL, Carvalho A dos S, and Pereira TS. Data analysis was performed by Menezes PFL, Carvalho A dos S, and Pereira TS. The analysis was supervised by Silva Junior JM, and Treml RE. The manuscript draft and its final version were written by Silva Junior JM, Treml RE, and Gurgel Segundo MR de M. All authors reviewed and approved the final manuscript. The person responsible for the study and corresponding author is Silva Junior JM who attests that all participants in this study meet the criteria for authorship.

All the authors declare that they have no conflicts of interest or financial interests related to this study.

## Figures and Tables

**Table 1:** Clinical and demographic data of the patients.

Variables	N (%)	Average ± SD	Median (IQR)
Age (years)	48 (100)	59.4±12.6	61.0 (52-66.7)
Sex			
Male	31 (65)	—	—
Female	17 (35)	—	—
BMI (kg/m^2^)	48 (100)	30.1±8.1	28.7 (24.5-32.9)
Comorbidities^1^			
Hypertension	26 (39.4)	—	—
Diabetes	23 (34.8)	—	—
Tobacco use	12 (18.2)	—	—
Chronic kidney disease	8 (12.1)	—	—
Asthma	4 (6.1)	—	—
Other	37 (56.1)	—	—
Time in prone position, hours	48 (100)	1.9±0.9	2.0 (1.0-3.0)
Intubated patients	16 (33.3)	—	—
Time to require endotracheal intubation after hospital admission (days)	16 (33.3)	4.63±2.9	5.0 (3.0-5.0)
Pulmonary involvement on CCT			
≤50%	18 (37)	—	—
>50%	30 (63)	—	—
LOS-ICU (days)	16 (33.3)	12.6±7.4	9.5 (7.0-18.0)
LOHS (days)	48 (100)	17.8±10.0	16.0 (10.0-22.0)
Hospital mortality	8 (16.7)	—	—

BMI, body mass index; CCT, computed tomography of the chest; IQR, interquartile range; LOS-ICU, length of stay in intensive care; LOHS, length of hospital stay; n, the absolute frequency of participants; SD, standard deviation;

1 Comorbidities: patients could present more than one.

**Table 2:** Comparison of patients in supine and prone position in patients with pulmonary involvement ≤50% and >50% evaluated by computed tomography of chest.

Variable	Pulmonary involvement < 50% (n=18)		*p*	Pulmonary involvement ≥ 50% (n=30)		*p*
	Supine	Prone		Supine	Prone	
pH			¢0.410			¢0.619
Average±SD	7.41±0.03	7.42±0.02		7.41±0.05	7.40±0.02	
Median (min; max.)	7.41 (7.30; 7.46)	7.41 (7.41; 7.49)		7.41 (7.22; 7.52)	7.41 (7.33; 7.46)	
PaO_2_ (mmHg)			¢0.006			¢0.680
Average±SD	73.2±10.5	83.6±6.8		87.6±33.8	84.7±16.4	
Median (min; max.)	82 (53.7; 83.6)	84.3 (64.4; 97.8)		82 (46.4; 207.5)	84.3 (62.4; 162.0)	
PaCO_2_ (mmHg)			¢0.518			¢0.002
Average±SD	39.5±6.1	40.6±3.0		40.5±6.0	42.9±4.9	
Median (min; max.)	40.2 (27.0; 56.4)	42.1 (31.5; 42.1)		40.2 (28.6; 58.9)	42.1 (33.0; 59.6)	
Arterial HCO_3_ (mEq/L)			¢0.373			¢0.001
Average±SD	25.1±2.5	25.7±1.6		25.4±2.5	26.5±2.2	
Median (min; max.)	25.3 (19.2; 28.9)	26.2 (20.3; 27.8)		25.3 (19.2; 32.2)	26.2 (20.4; 32.7)	
SaO_2_ (%)			¢ < 0.001			¢0.023
Average±SD	92.7±1.4	95.3±0.8		93.2±3.4	94.8±1.1	
Median (min; max.)	93 (90.0; 96.0)	95 (95.0; 98.0)		93 (83.0; 100)	95 (92; 98)	
SpO_2_ (%)			¢0.006			0.013**
Average±SD	90.8±5.7	94.7±1.9		91.2±6.7	95.6±2.3	
Median (min; max.)	92 (70; 96)	95 (91; 95)		92 (74; 99)	96 (92; 99)	
Respiratory rate			¢0.547			0.238**
Average±SD	20.9±4.1	20.1±3.0		23.1±4.5	22.0±3.7	
Median (min; max.)	20 (16; 34)	20 (12; 26)		22 (16; 34)	22 (14; 32)	
O_2_ flow (L/min)			0.294**			¢0.101
Average±SD	5.5±3.7	4.6±2.3		7.0±3.9	6.4±4.1	
Median (min; max.)	5 (1; 15)	5 (1; 10)		6 (2; 15)	5 (1; 15)	
Respiratory support, n (%)			1.000*			1.000*
Nasal catheter	10 (55.6)	10 (55.6)		12 (40.0)	12 (40.0)	
NIV (HFNC or CPAP)	0 (0.0)	0 (0.0)		7 (23.3)	7 (23.3)	
Nonrebreather mask	8 (44.4)	8 (44.4)		11 (36.7)	11 (36.7)	
Heart rate(bpm)			0.705**			0.243**
Average±SD	86.6±15.2	88.2±13.3		83.9±13.4	80.9±15.2	
Median (min; max.)	82 (65; 120)	86 (68; 116)		85 (60; 120)	80 (52; 103)	
Arterial lactate (mmol/L)			¢0.372			¢0.031
Average±SD	2.0±0.5	1.87±0.3		2.13±0.62	1.87±0.05	
Median (min; max.)	2.1 (1.1; 3.2)	1.9 (0.9; 2.4)		2.1 (1.4; 5.2)	1.9 (1.7; 2.0)	
Mean blood pressure (mmHg)			0.212**			¢0.010
Average±SD	96.0±117	100.5±10.5		94.0±13.8	100.3±14.2	
Median (min; max.)	97 (60; 117)	100 (77; 122)		93 (73; 134)	98 (77; 146)	

HCO_3_: arterial bicarbonate; PaCO_2_: partial pressure of carbon dioxide; PaO_2_ **:** arterial partial pressure of oxygen; SaO_2_: arterial oxygen saturation; SpO_2_: peripheral oxygen saturation; SD: standard deviation.

* Fisher's exact test;

** T-test of paired samples; ¢ Wilcoxon signed-rank test (paired samples).
